# Clinical and laboratory markers to distinguish VEXAS from Schnitzler's syndrome: data from the AIDA network registries

**DOI:** 10.3389/fmed.2025.1659758

**Published:** 2026-01-12

**Authors:** Valeria Caggiano, Jessica Sbalchiero, Micol Frassi, Eduardo Martín-Nares, Andrea Hinojosa-Azaola, Mariusz Sikora, Karina Jahnz-Różyk, Francesca Crisafulli, Franco Franceschini, Paolo Airò, Guillermo Arturo Guaracha-Basañez, Jiram Torres-Ruiz, Paolo Sfriso, Sara Bindoli, Chiara Baggio, José Hernández-Rodríguez, Verónica Gómez Caverzaschi, Gerard Espinosa, Henrique A. Mayrink Giardini, Rafael Alves Cordeiro, Andrés González-García, Mercedes Peña Rodríguez, Giuseppe Lopalco, Florenzo Iannone, Ombretta Viapiana, Abdurrahman Tufan, Hamit Kucuk, Pravin Hissaria, Mark Beecher, Amato De Paulis, Ilaria Mormile, Lorenzo Dagna, Corrado Campochiaro, Antonio Gidaro, Leyla La Cava, Serena Bugatti, Alessandra Milanesi, Guillermo Ruiz-Irastorza, Matteo Piga, Fabrizio Conti, Paolo Moscato, Daniela Opris-Belinski, Rosetta Vitetta, Cecilia Chighizola, Andreas Recke, Fernando Tornero-Romero, Marcella Prete, Marcello Govoni, Giacomo Emmi, Perla Ayumi Kawakami-Campos, Paola Triggianese, Carmelo Gurnari, Gaafar Ragab, Alberto Balistreri, Marcin Ziȩtkiewicz, Ewa Wiesik-Szewczyk, Bruno Frediani, Claudia Fabiani, Anna Sicuranza, Monica Bocchia, Luca Cantarini, Antonio Vitale

**Affiliations:** 1 Department of Medical Sciences, Surgery and Neurosciences, Research Center of Systemic Autoinflammatory Diseases and Behçet's Disease Clinic, University of Siena, Siena, Italy; 2 Azienda Ospedaliero-Universitaria Senese [European Reference Network (ERN) for Rare Immunodeficiency, Autoinflammatory and Autoimmune Diseases (RITA) Center], Siena, Italy; 3 Rheumatology and Clinical Immunology, Spedali Civili and Department of Clinical and Experimental Sciences, University of Brescia, [European Reference Network (ERN) for Rare Immunodeficiency, Autoinflammatory and Autoimmune Diseases (RITA) Center], Brescia, Italy; 4 Department of Immunology and Rheumatology, Instituto Nacional de Ciencias Médicas y Nutrición Salvador Zubirán, Mexico City, Mexico; 5 Department of Internal Medicine, Pneumonology, Allergology, Clinical Immunology and Rare Diseases, Military Institute of Medicine, National Research Institute, Warsaw, Poland; 6 Rheumatology Unit, Department of Medicine, University of Padua, [European Reference Network (ERN) for Rare Immunodeficiency, Autoinflammatory and Autoimmune Diseases (RITA) Center], Padua, Italy; 7 Clinical Unit of Autoinflammatory Diseases, Department of Autoimmune Diseases, Institut d'Investigacions Biomèdiques August Pi I Sunyer (IDIBAPS), Hospital Clínic of Barcelona [European Reference Network (ERN) for Rare Immunodeficiency, Autoinflammatory and Autoimmune Diseases (RITA) Center], University of Barcelona, Barcelona, Spain; 8 Rheumatology Division, Faculdade de Medicina, Hospital das Clínicas, Universidade de São Paulo, São Paulo, Brazil; 9 Systemic Autoimmune Diseases Unit, Department of Internal Medicine, Hospital Universitario Ramón y Cajal, IRYCIS, Madrid, Spain; 10 Department of Precision and Regenerative Medicine and Ionian Area (DiMePRe-J) Policlinic Hospital, University of Bari, Bari, Italy; 11 Rheumatology Unit, Department of Medicine, University and Azienda Ospedaliera Universitaria Integrata of Verona, Verona, Italy; 12 Department of Internal Medicine, Division of Rheumatology, Gazi University Hospital, Ankara, Türkiye; 13 Department of Clinical Immunology and Allergy, Royal Adelaide Hospital, Adelaide, SA, Australia; 14 Department of Immunopathology, SA Pathology, Adelaide, SA, Australia; 15 Department of Translational Medical Sciences, Section of Clinical Immunology, University of Naples Federico II, Naples, Italy; 16 Center for Basic and Clinical Immunology Research (CISI), WAO Center of Excellence, University of Naples Federico II, Naples, Italy; 17 Division of Immunology, Transplants and Infectious Diseases, Università Vita-Salute San Raffaele, Milan, Italy; 18 Unit of Immunology, Rheumatology, Allergy and Rare Diseases, IRCCS Ospedale San Raffaele, [European Reference Network (ERN) for Rare Immunodeficiency, Autoinflammatory and Autoimmune Diseases (RITA) Center], Milan, Italy; 19 Department of Biomedical and Clinical Sciences Luigi Sacco, Luigi Sacco Hospital, University of Milan, Milan, Italy; 20 Department of Internal Medicine and Therapeutics, Università di Pavia, Pavia, Italy; 21 Division of Rheumatology, Fondazione IRCCS Policlinico San Matteo, [European Reference Network (ERN) for Rare Immunodeficiency, Autoinflammatory and Autoimmune Diseases (RITA) Center], Pavia, Italy; 22 Faculty of Medicine and Nursery, University of the Basque Country, UPV/EHU, Leioa, Biscay, Spain; 23 Autoimmune Diseases Unit, Biocruces Bizkaia Health Research Institute, Barakaldo, Spain; 24 Rheumatology Unit, Department of Medical Sciences, University and AOU of Cagliari, Cagliari, Italy; 25 Department of Internal Medicine and Medical Specialties, Rheumatology Unit, AOU Policlinico Umberto I, Sapienza University of Rome, Rome, Italy; 26 UOC of Internal Medicine - Rheumatology Outpatients Unit, Azienda Ospedaliero-Universitaria San Giovanni di Dio e Ruggi D'Aragona, Salerno, Italy; 27 Rheumatology and Internal Medicine Department, Carol Davila University of Medicine and Pharmacy, Bucharest, Romania; 28 Unit of Rheumatology, ASL VC Sant' Andrea Hospital, Vercelli, Italy; 29 Department of Clinical Sciences and Community Health, Research Center for Adult and Pediatric Rheumatic Diseases, University of Milan, Milan, Italy; 30 Department of Dermatology, Allergology and Venerology, University Hospital Schleswig-Holstein, Lübeck, Germany; 31 Autoinflammatory and Autoimmune Diseases (RITA) Center, European Reference Network (ERN) for Rare Immunodeficiency, Lübeck, Germany; 32 Medicina Interna - Enfermedades Autoinmunes Sistémicas, Hospital Fundación Jiménez Díaz, Madrid, Spain; 33 Rheumatic and Systemic Autoimmune Diseases Unit, Department of Interdisciplinary Medicine (DIM), University of Bari Medical School, Bari, Italy; 34 Rheumatology Unit, Department of Medical Sciences, Azienda Ospedaliero-Universitaria S. Anna-Ferrara, University of Ferrara, Ferrara, Italy; 35 Department of Medical, Surgical and Health Sciences, University of Trieste, Trieste, Italy; 36 Clinical Medicine and Rheumatology Unit, Cattinara University Hospital, Trieste, Italy; 37 Centre for Inflammatory Diseases, Department of Medicine, Monash Medical Centre, Monash University, Clayton, VIC, Australia; 38 Department of Ophthalmology, Instituto Nacional de Ciencias Médicas y Nutrición Salvador Zubirán, Mexico City, Mexico; 39 UOC Medicina Interna - UOSD Geriatria, Università di Roma Tor Vergata, Rome, Italy; 40 Department of Biomedicine and Prevention, University of Rome Tor Vergata, Rome, Italy; 41 Department of Translational Hematology and Oncology Research, Taussig Cancer Institute, Cleveland Clinic, Cleveland, OH, United States; 42 Internal Medicine Department, Rheumatology and Clinical Immunology Unit, Faculty of Medicine, Cairo University, Giza, Egypt; 43 Faculty of Medicine, Newgiza University, 6th of October City, Egypt; 44 Bioengineering and Biomedical Data Science Lab, Department of Medical Biotechnologies, University of Siena, Siena, Italy; 45 Department of Rheumatology, Clinical Immunology, Geriatrics and Internal Medicine, Medical University of Gdansk, Gdansk, Poland; 46 Ophthalmology Unit, Department of Medicine, Surgery and Neurosciences, University of Siena, Siena, Italy; 47 Hematology, Azienda Ospedaliera Universitaria Senese, University of Siena, Siena, Italy

**Keywords:** UBA1 mutation, autoinflammatory diseases, cutaneous manifestations, differential diagnosis, treatment

## Abstract

**Background:**

A substantial overlap in demographic, clinical, and laboratory features can complicate the differential diagnosis between Schnitzler's syndrome and VEXAS syndrome. The present study was undertaken to identify clinical and laboratory parameters that should raise suspicion for VEXAS syndrome among patients previously diagnosed with, or under evaluation for, Schnitzler's syndrome.

**Methods:**

Data from male-only patients with Schnitzler's syndrome or VEXAS syndrome were obtained from international AIDA Network registries. Subjects with Schnitzler's syndrome were compared to VEXAS patients with urticarial skin manifestations resembling cutaneous features typically observed in Schnitzler's syndrome.

**Results:**

A total of 19 VEXAS patients and 18 patients with Schnitzler's syndrome were enrolled. At univariate binary logistic regression, the diagnosis of VEXAS syndrome was associated with the age at disease onset (OR = 1.08, 95% CI. 1.01–1.16, *p* = 0.02), hemoglobin levels (OR = 0.44, 95% CI. 0.26–0.77, *p* = 0.003), anemia (OR = 13.9, 95% CI. 3.4–5.7, *p* = 0.02), leucocytosis (OR = 0.04, 95% CI. 0.06–0.22, *p* < 0.001), lymphadenopathy (OR = 7.8, 95% CI. 1.41–45.4, *p* = 0.02), and thrombocytopenia (OR = 13.5, 95% CI. 1.47–123.7, *p* = 0.02). In the multivariable logistic regression analysis with the stepwise forward selection approach, the diagnosis of VEXAS syndrome was significantly associated with the age at disease onset (OR: 1.13, 95% CI: 1.02–1.30, *p* = 0.04) and the presence of lymphadenopathy (OR: 67.49, 95% CI: 5.36–3284.89, *p* = 0.007), while thrombocytopenia showed a trend toward statistical significance (OR: 12.02, 95% CI: 1.07–315.86, *p* = 0.06).

**Conclusions:**

Patients with lymphadenopathy, thrombocytopenia, anemia, particularly in older age and in the absence of leucocytosis, are more likely to be affected by VEXAS syndrome rather than Schnitzler's syndrome.

## Introduction

Vacuoles, Enzyme-1, X-linked, Autoinflammatory, Somatic (VEXAS) syndrome is a recently described adult-onset systemic autoinflammatory disorder, first identified by Beck et al. (1). This syndrome represents a novel paradigm among autoinflammatory diseases, characterized by a complex clinical presentation driven by somatic mosaicism of *UBA1* gene mutations within the framework of an X-linked genetic pattern. The diagnosis is often challenging due to its broad spectrum of manifestations, overlapping with numerous other inflammatory and autoimmune conditions, including systemic vasculitis, many forms of arthritis, as seronegative arthritis or spondyloarthritis, along with polymyalgia rheumatica, Behçet's disease, Sweet's syndrome, and relapsing polychondritis (2–5). The VEXAS syndrome may also involve the hematologic compartment especially with macrocytic anemia, thrombocytopenia, lymphocytopenia, myelodysplastic syndrome and monoclonal gammopathy of undetermined significance (MGUS) (3, 6).

Along with other inflammatory manifestations, cutaneous involvement is a common feature of VEXAS syndrome, frequently presenting as neutrophilic dermatoses resembling Sweet's syndrome or urticarial lesions that mimic the dermatological features of other autoimmune and autoinflammatory disorders (4). Among these, Schnitzler's syndrome represents a particularly relevant differential diagnosis. Schnitzler's syndrome is a rare, multifactorial autoinflammatory condition characterized by a chronic urticarial skin rash, intermittent fever, bone and/or joint pain, lymphadenopathy, and monoclonal gammopathy of undetermined significance (MGUS), often, but not exclusively, of the IgM type, as IgG variants are also relatively common (7, 8). Unlike in VEXAS syndrome, the molecular basis of inflammation in Schnitzler's syndrome remains unclear. The specific *MYD88* L265P mutation, which encodes an adaptor protein involved in Toll-like receptor and interleukin-1 receptor (IL-1R) signaling pathways (9), has been identified in approximately 30% of patients, and clonal hematopoiesis has been observed in only a few cases of Schnitzler's syndrome (10). Similar to VEXAS syndrome, Schnitzler's syndrome typically affects middle-aged to older adults and may present with overlapping features, especially urticarial skin rash, unexplained systemic inflammation, and the presence of a MGUS as an expression of hematologic involvement (11). While in Schnitzler's syndrome the male-to-female ratio ranges from 1.4:1 to 1.76:1, indicating a slight male predominance in most reported series (11, 12), VEXAS syndrome shows a marked male predominance, with an estimated prevalence of 1 in 4,269 among men and 1 in 26,238 among women, corresponding to a ratio of ~1:6. However, clinical cohorts and case series consistently report even higher ratios, typically in the range of 1:25 to 1:30 (13).

Currently, the optimal treatment strategy for VEXAS syndrome is still to be defined. While the chronic use of glucocorticoids, at low-to-high dosages according to the severity of the clinical picture, has proven useful in controlling the clinical and laboratory manifestations of the disease, anti-IL-1 and anti-IL-6 agents yield very heterogeneous results, as do Janus kinase (JAK) inhibitors, which have shown some encouraging results, especially when JAK2 is more specifically targeted (14–16). Conversely, Schnitzler's syndrome has been shown to respond completely to anti-IL-1 agents in the majority of cases, while IL-6 inhibition represents a promising therapeutic approach. Therefore, both VEXAS and Schnitzler's syndromes are frequently managed with IL-1 and IL-6 inhibitors, constituting commonly employed treatment strategies in the broader context of autoinflammatory diseases (15–20). This additional overlap in treatment further contributes to the complexity of the differential diagnosis between VEXAS syndrome and Schnitzler's syndrome and underscores the similarities between these two conditions within the spectrum of autoinflammatory disorders.

In light of the substantial clinical, demographic, and therapeutic overlap between these two entities, patients diagnosed with Schnitzler's syndrome, particularly in male cases, may represent misdiagnosed cases of VEXAS syndrome. Therefore, precise differential diagnosis is imperative to avoid misclassification, allow for more accurate risk stratification and long-term outcome prediction, and to guide optimal therapeutic strategies. In this context, the present study seeks to characterize the distinguishing features between VEXAS syndrome and Schnitzler's syndrome through the systematic analysis of clinical and laboratory parameters in a defined cohort of male patients presenting with cutaneous involvement. This investigation leverages real-world data collected within the framework of the International AutoInflammatory Disease Alliance (AIDA) Network registries specifically dedicated to VEXAS syndrome and Schnitzler's syndrome (21, 22).

## Materials and methods

Patients with VEXAS syndrome were consecutively enrolled between November 2021 and April 2025 in the international AIDA Network registry dedicated to VEXAS syndrome (21). Patients with Schnitzler's syndrome were enrolled during the same period in the AIDA Network registry dedicated to Schnitzler's syndrome (22). Data collection was retrospective and included clinical and laboratory information from disease onset up to the time of enrollment in the AIDA registry. All VEXAS patients included in this study presented skin involvement, characterized either by isolated urticarial lesions or by urticarial lesions in combination with other cutaneous manifestations. All Schnitzler's and VEXAS patients included in this study were males; genetic analyses were performed in all patients with Schnitzler's syndrome included in the study to rule out *UBA1* gene mutations that might explain the observed clinical phenotype. Only male patients were enrolled in the study, as the differential diagnosis between VEXAS and Schnitzler's syndrome predominantly concerns male patients due to the marked male predominance of VEXAS syndrome, at current. Female cases are extremely rare, with up to 12 described worldwide (23), and only one female patient in the AIDA registry fulfilling the inclusion criteria. Therefore, the study was restricted to male subjects to ensure a homogeneous and representative cohort.

The primary objective of this study is to evaluate the clinical and laboratory differences between male patients with Schnitzler's syndrome and those with VEXAS syndrome presenting with cutaneous involvement. By analyzing patients with similar demographic and dermatological features, the study aimed to enhance diagnostic accuracy and support the clinical differentiation between these two autoinflammatory conditions. Additional objectives include the identification of clinical and laboratory variables significantly associated with the diagnosis of VEXAS syndrome and that may assist clinicians in differential diagnosis in clinical practice.

Inclusion criteria for patients affected by VEXAS syndrome required the presence of a pathogenic or likely pathogenic mutation in the *UBA1* gene, along with the onset of a systemic inflammatory condition not otherwise explained. For patients with Schnitzler's syndrome, the inclusion criteria required the fulfillment of Strasbourg diagnostic criteria for Schnitzler's syndrome (11). Additionally, patients were required to provide signed informed consent for the use of clinical, laboratory, and genetic data within the AIDA Network. The study was approved by the Ethics Committee of the Azienda Ospedaliero-Universitaria Senese, Siena, Italy in June 2019 (Ref. N. 14951) as part of the AIDA Program. The study protocol conformed to the tenets of the Declaration of Helsinki.

Mutations in the *UBA1* gene were detected through Next Generation Sequencing or Sanger testing, performed on peripheral blood or bone marrow samples obtained from patients. Given the multicentric nature of the registry, sequencing and bioinformatic analyses were conducted according to each centre's validated protocols, with all identified variants subsequently harmonized and centrally re-annotated to ensure methodological consistency across the cohort.

Among clinical features, arthritis was defined by the presence of at least one swollen joint or ultrasound-confirmed synovitis in at least one joint, with Doppler activity also assessed during the examination. Gastrointestinal involvement referred to the presence of abdominal pain with or without diarrhea. Ocular involvement included episcleritis, uveitis, scleritis, blepharitis, periorbital edema, conjunctival chemosis, and eyelid swelling. Vascular involvement included stroke, critical limb-threatening ischemia, bowel infarction, pulmonary embolism, and deep vein thrombosis. Renal involvement was defined by the presence of proteinuria, erythrocyturia with dysmorphic erythrocytes, and progressive renal impairment up to kidney failure. Neurological involvement encompassed minor or major cerebrovascular events, meningitis, and peripheral nervous system manifestations such as sensory neuropathy and multiple mononeuropathy.

Laboratory data included the identification of anemia, leukopenia, leukocytosis, thrombocytosis, and thrombocytopenia at the time of disease onset. Anemia was defined as hemoglobin (Hb) ≤ 12 g/dL; leukocytosis as a white blood cell (WBC) count >15,000/μL; and leukopenia as a WBC count < 4,000/μL. A platelet count ≥450,000/μL was defined as thrombocytosis, whereas a count ≤ 150,000/μL was considered thrombocytopenia (24–27).

Regarding treatment outcomes, the response criteria established by the AIDA Network were applied to ensure consistency across studies (14). In particular, *complete response* was defined as the resolution of all disease-related clinical manifestations, along with the normalization of all inflammatory laboratory parameters. *Partial response* was characterized by the persistence of clinical manifestations, with a significant reduction in their severity, as reported by patients and observed by physicians, and with inflammatory laboratory markers either normalized or only slightly increased above normal. *Treatment Failure* was defined by the persistence of clinical manifestations and/or the absence of a decrease in laboratory inflammatory markers.

Regarding statistical computations, descriptive statistics included percentages, means, standard deviation (SD), median and interquartile range (IQR), and frequency counts, as required. Qualitative data were analyzed with the Fisher Exact test or the Chi squared test based on frequency distribution and the expected frequency counts. Quantitative variables were analyzed by using Welch two-sample test or Mann-Whitney U test according to data distribution assessed with the Shapiro-Wilk test. Subsequently, a univariate binomial logistic regression analysis was conducted to evaluate potential associations between the diagnosis of VEXAS syndrome and those variables that had significantly discriminated patients with VEXAS syndrome from those with Schnitzler's syndrome in the preceding inferential analyses. Thereafter, variables that showed a statistically significant association in the univariate analysis were entered into a multivariable logistic regression model using a stepwise forward selection approach, in order to identify independent predictors of VEXAS syndrome diagnosis. Significance level was set at 95% (*p*-value < 0.05); the *p*-value was two-tailed. Statistical analysis was plotted through the RStudio software, version 4.3.0.

## Results

A total of 19 patients affected by VEXAS syndrome and 18 with Schnitzler's syndrome were included in this study. Specific clinical features and laboratory data of the VEXAS syndrome and Schnitzler's syndrome groups are reported in Table 1. The mean age at disease onset was 64.1 ± 12.5 years in the VEXAS group and 53.55 ± 11.2 years among patients affected by Schnitzler's syndrome (*p* = 0.01). The mean age at diagnosis was 66.8 ± 10.1 years in the VEXAS group and 56.6 ± 12.1 years in the Schnitzler's group (*p* = 0.01). Table 2 details the *UBA1* mutations observed in the patients with VEXAS syndrome.

**Table 1:** Specific clinical manifestations and laboratory features observed in patients with VEXAS syndrome and those in the Schnitzler's syndrome group.

**VEXAS syndrome, 19 patients**	
Fever, *n* (%)	16 (84.2)
Temperature max (°C), mean ± SD	38.9 ± 1.17
Orbital/Ocular involvement, *n* (%)	9 (47.4)
Weight loss, *n* (%)	17 (89.5)
Arthritis, *n* (%)	5 (26.3)
Arthralgia, *n* (%)	16 (84.2)
Myalgia, *n* (%)	10 (52.6)
Gastrointestinal involvement, *n* (%)	5 (26.3)
Splenomegaly, *n* (%)	4 (21.1)
Neurological involvement, *n* (%)	1 (5.3)
Vascular involvement, *n* (%)	9 (47.4)
Kidney involvement, *n* (%)	1 (5.3)
Lymphadenopathy, *n* (%)	9 (47.4)
Thoracic pain, *n* (%)	2 (10.5)
Pleuritis, *n* (%)	1 (5.3)
Pericarditis, *n* (%)	1 (5.3)
Recurrent polychondritis	1 (5.3)
Anemia, *n* (%) •*Macrocytic, n (%)* •*Normocytic, n (%)*	18 (94.7) 16 (88.9) 2 (11.1)
Hemoglobin level (g/dL), median (IQR)	9.4 (3.15)
Leukopenia, *n* (%) •*Lymphopenia, n (%)* •*Neutropenia, n (%)* •*Monocytopenia, n (%)*	10 (52.6) 7 (70) 7 (70) 1 (10)
Leukocytosis, *n* (%)	2 (10.5)
Thrombocytopenia, *n* (%)	9 (47.4)
**Schnitzler's syndrome, 18 patients**	
Fever, *n* (%)	18 (100)
Temperature max (°C), mean ± SD	38.9 ± 0.63
Ocular involvement, *n* (%)	1 (5.5)
Thoracic pain, *n* (%)	2 (11.1)
Lymphadenopathy, *n* (%)	2 (11.1)
Splenomegaly, *n* (%)	2 (11.1)
Myalgia, *n* (%)	14 (77.8)
Arthralgia, *n* (%)	16 (88.9)
Arthritis, *n* (%)	2 (11.1)
Bone pain, *n* (%)	5 (27.8)
Aseptic osteomyelitis, *n* (%)	3 (16.7)
Gastrointestinal involvement, *n* (%)	2 (11.1)
Neurological involvement, *n* (%)	2 (11.1)
Anemia, *n* (%)	9 (81.8)
Hemoglobin level (g/dL), median (IQR)	14.4 (2.3)
Neutrophilia, *n* (%)	13 (72.2)
Lymphopenia, *n* (%)	6 (33.3)
Thrombocytosis, *n* (%)	5 (27.8)
Thrombocytopenia, *n* (%)	1 (5.5)
Increased serum Immunoglobulins G, *n* (%)	3 (16.7)
Increased serum Immunoglobulins M, *n* (%)	9 (81.8)

**Table 2:** Mutations identified in the *UBA1* gene among patients with VEXAS syndrome.

**Specific mutations in *UBA1* gene**	***n* (%)**
M41L (p.Met41Leu)	2 (10.5)
M41T (p.Met41Thr)	9 (47.4)
M41V (p.Met41Val)	7 (36.8)
p.(Ser56Phe)	1 (5.3)

All patients exhibited skin involvement, either with isolated urticarial lesions or in combination with other cutaneous manifestations. Specifically, isolated urticarial lesions were observed in 20 (54.1%) patients, including 8 (40%) in the VEXAS group and 12 (60%) in the Schnitzler's group. Urticarial lesions associated with additional cutaneous features were detected in 17 (45.9%) patients. In particular, erythematous lesions were reported in 5 (29.4%) patients with VEXAS syndrome and 3 (17.6%) patients with Schnitzler's syndrome (*p* = 0.69); maculopapular lesions were observed in 4 (23.5%) VEXAS patients and 3 (17.6%) Schnitzler's patients (*p* = 1.0); pustular manifestations were found in 3 (17.6%) VEXAS patients and in none of the Schnitzler's patients (*p* = 1.0); and neutrophilic dermatoses were detected in 6 (35.3%) VEXAS patients and 2 (11.8%) Schnitzler's patients (*p* = 0.23).

Among clinical manifestations lymphadenopathy emerged as a relevant discriminating clinical feature, being reported in 9 (47.4%) patients with VEXAS syndrome compared to 2 (11.1%) patients in the Schnitzler's group (*p* = 0.03).

Concerning hematologic laboratory data, anemia was observed in 18 (94.7%) patients in the VEXAS group and in 9 (50%) subjects with Schnitzler's syndrome (*p* = 0.013). Median hemoglobin levels were 9.4 g/dl (IQR 3.15) in the VEXAS group and 14.4 g/dL (IQR 2.3) in the Schnitzler's group (*p* < 0.001). Leukocytosis was detected in 2 (10.5%) and 13 (72.2%) patients affected by VEXAS and Schnitzler's syndrome (*p* < 0.001), respectively, while leukopenia was observed in 10 (52.6%) patients of VEXAS group and in no cases in the Schnitzler group (*p* < 0.001). Thrombocytosis was identified in 5 (27.8%) patients with Schnitzler's syndrome and in none of the VEXAS subjects (*p* = 0.013); thrombocytopenia was detected in 9 (47.4%) subjects with VEXAS syndrome and in one patient (5.6%) affected by Schnitzler's syndrome (*p* = 0.01).

Regarding concomitant paraproteinemia, MGUS was present in all patients with Schnitzler's syndrome and in 3 (15.8%) patients with VEXAS syndrome (*p* < 0.001). In addition, myelodysplastic syndromes were identified in 10 (52.6%) patients with VEXAS syndrome, whereas no cases were observed among patients with Schnitzler's syndrome (*p* < 0.001).

At univariate binary logistic regression, the diagnosis of VEXAS syndrome was associated with the age at disease onset (OR = 1.08, 95% CI. 1.01–1.16, *p* = 0.02), hemoglobin levels (OR = 0.44, 95% CI. 0.26–0.77, *p* = 0.003), the presence of anemia (OR = 13.9, 95% CI. 3.4–5.7, *p* = 0.02), leucocytosis (OR = 0.04, 95% CI. 0.06–0.22, *p* < 0.001), lymphadenopathy (OR = 7.8, 95% CI. 1.41–45.4, *p* = 0.02), and thrombocytopenia (OR = 13.5, 95% CI. 1.47–123.7, *p* = 0.02).

**Figure 1:**
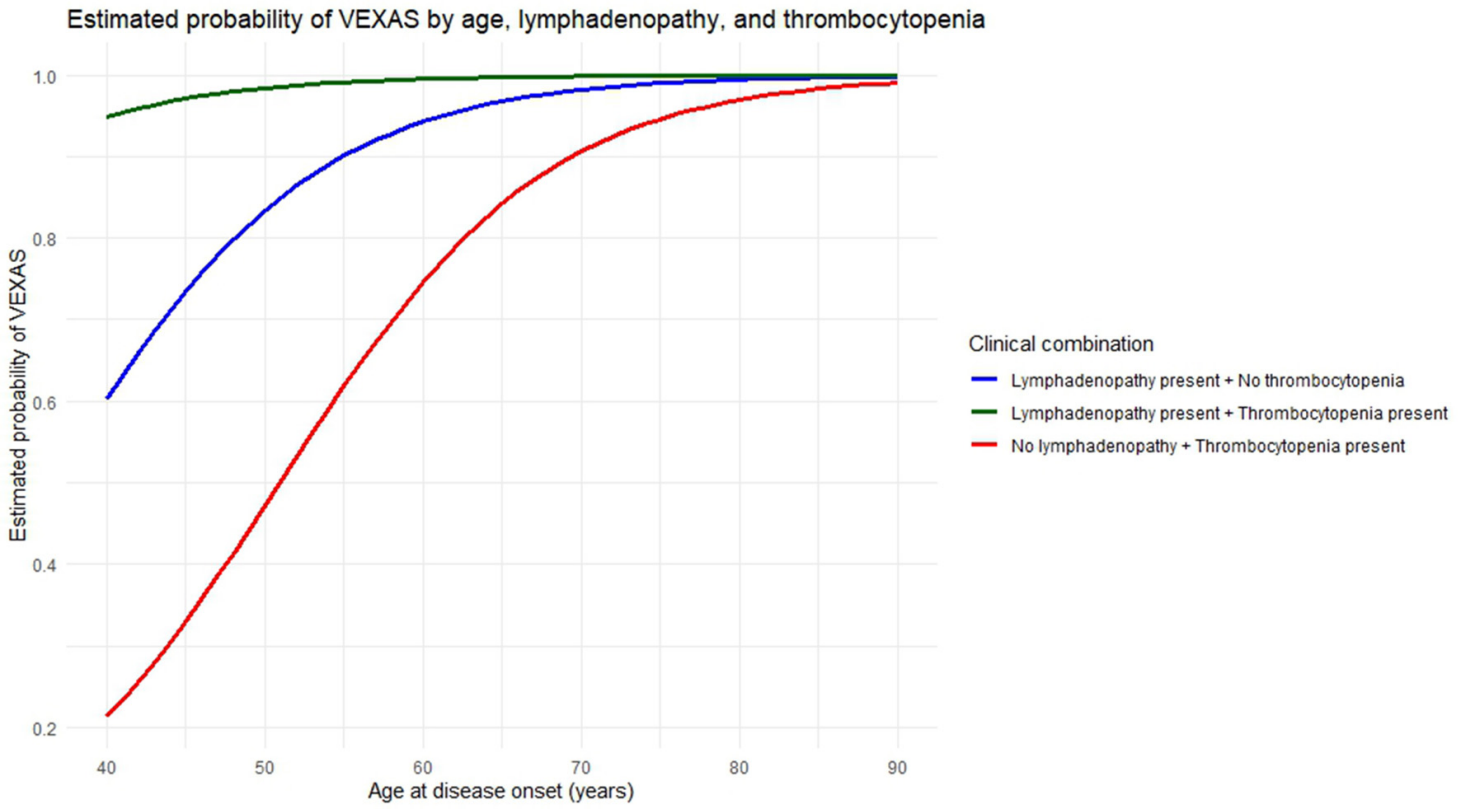
Predicted probability that a patient with clinical features overlapping between VEXAS syndrome and Schnitzler's syndrome actually suffers from VEXAS syndrome. The figure illustrates the estimated probability of a VEXAS syndrome diagnosis as a function of age at disease onset, according to three distinct clinical profiles defined by the presence or absence of lymphadenopathy and thrombocytopenia. The green curve corresponds to individuals presenting both lymphadenopathy and thrombocytopenia, indicating a high probability of VEXAS syndrome across all ages. The blue curve represents individuals with lymphadenopathy but without thrombocytopenia, with probabilities increasing gradually with age. The red curve refers to individuals with thrombocytopenia in the absence of lymphadenopathy, displaying lower estimated probabilities at younger ages, which increase progressively with advancing age. Note that 95% confidence intervals were omitted for the three expected probability curves, as the small sample size led to wide, overlapping intervals that could reduce clarity and interpretability.

In the multivariable logistic regression analysis with the stepwise forward selection approach, the diagnosis of VEXAS syndrome was significantly associated with the age at disease onset (OR: 1.13, 95% CI: 1.02–1.30, *p* = 0.04), the presence of lymphadenopathy (OR: 67.49, 95% CI: 5.36–3,284.89, *p* = 0.007), and thrombocytopenia (OR: 12.02, 95% CI: 1.07–315.86, *p* = 0.06). Figure 1 shows the probability of a VEXAS diagnosis based on age at disease onset, in the presence or absence of the other two variables.

Concerning treatment, 2 (10.5%) VEXAS patients received anti-IL1 biotechnological agents, with one showing partial response and one a treatment failure. On the other hand, 17 (94.4%) patients with Schnitzler's syndrome received this class of treatment, resulting in a complete response in 15 (88.2%) cases and a partial response in 2 (11.8%) patients. Additionally, 3 (16.7%) patients in the Schnitzler group and 3 (15.8%) subjects with VEXAS syndrome were treated with anti-IL6. All patients with Schnitzler's syndrome presented a partial response, while in the VEXAS group, 2 (66.7%) patients showed a partial response, and 1 (33.3%) patient experienced treatment failure.

## Discussion

The recognition of newly defined, genetically determined disease entities highlights the need to re-evaluate patients previously diagnosed with undifferentiated autoinflammatory syndromes or multifactorial autoinflammatory conditions which, in light of recent advances, may in fact represent previously unrecognized but now better-characterized disorders. This is particularly relevant for patients with Schnitzler's syndrome, a condition in which clonal hematopoiesis has been reported in a few cases and a potential association with the *MYD88* gene, involved in Toll-like receptor and IL-1 receptor signaling dysregulation, has been suggested (10). Indeed, the clinical phenotype of Schnitzler's syndrome may closely resemble that of VEXAS syndrome. Notably, some of these patients may carry pathogenic *UBA1* mutations and therefore be affected by this recently identified clinical entity. To explore this hypothesis, we selected male patients with Schnitzler's syndrome and compared them with individuals diagnosed with VEXAS syndrome who exhibited urticarial-like cutaneous manifestations consistent with the clinical features of Schnitzler's syndrome. Therefore, based on the comparative analysis conducted in this study, we identified key clinical and laboratory differences between male patients with Schnitzler's syndrome and those with VEXAS syndrome presenting with skin manifestations. These findings contribute to a clearer delineation between the two syndromes, which is essential for improving diagnostic accuracy and guiding appropriate management strategies.

Noteworthy, age at disease onset emerged as a key discriminating factor, as the likelihood of VEXAS syndrome appears to increase with advancing age. This finding supports the concept that *UBA1* mutations in hematopoietic progenitor cells are part of the immunoaging process (3, 28, 29), which explains why VEXAS syndrome predominantly affects older individuals (4). Indeed, *UBA1* mutations arise somatically in hematopoietic stem cells, contributing to a shift toward myelopoiesis and acquisition of senescence-like programs, with consequent impaired age-related clonal hematopoiesis and decline of immune function (30, 31).

According to the data from the present study, among the clinical features analyzed lymphadenopathy was found to be associated with VEXAS syndrome. This is a noteworthy finding, given that Schnitzler's syndrome itself has a hematologic origin and is frequently accompanied by lymphadenomegaly, with reported prevalence ranging from 21% to 44% across different cohorts (32–34). In the current male-only cohort, lymphadenopathy was scarcely represented among patients with Schnitzler's syndrome, whereas it was observed in approximately half of the VEXAS patients. This suggests that in VEXAS syndrome, despite its known association with myelodysplastic syndrome, MGUS and lymphadenopathy (4), the presence of a pronounced systemic inflammatory component may promote lymph node enlargement to an even greater extent than is typically observed in Schnitzler's syndrome.

Thrombocytopenia represents another key feature associated with VEXAS syndrome, consistently reported as a hallmark finding of this condition. Actually, thrombocytopenia has been widely reported in VEXAS syndrome and has even been identified as a distinguishing laboratory marker capable of differentiating VEXAS patients among those presenting with relapsing polychondritis (6, 35). For this reason, although multivariable analysis revealed only a trend toward statistical significance, the well-established relevance of thrombocytopenia in the literature and its substantial clinical value justified its inclusion in the final predictive model.

In general, laboratory findings appear to offer superior discriminatory power compared to clinical features when distinguishing between VEXAS syndrome and Schnitzler's syndrome. Specifically, univariate regression analysis showed that the presence of anemia and lower hemoglobin levels significantly increase the likelihood of a VEXAS syndrome diagnosis, whereas leukocytosis is associated with a reduced probability. Conversely, the higher prevalence of MGUS among Schnitzler's syndrome patients is merely a reflection of the Strasbourg criteria, which require a monoclonal gammopathy for diagnosis, an element that is not necessary for the diagnosis of VEXAS syndrome (11).

Regarding the role of laboratory manifestations, multiple mechanisms likely underlie the high prevalence of anemia and thrombocytopenia observed in VEXAS syndrome, reflecting the complex interplay between clonal hematopoietic dysfunction, bone marrow dysplasia, and chronic inflammation. Somatic mutations in the *UBA1* gene, occurring in hematopoietic stem and progenitor cells, give rise to clonal expansion of dysfunctional myeloid cells that progressively dominate hematopoiesis, leading to ineffective blood cell production and cytopenias such as anemia and thrombocytopenia (6, 36, 37). Bone marrow findings in affected patients typically reveal hypercellularity with myeloid hyperplasia, erythroid hypoplasia, and characteristic vacuolization of myeloid and erythroid precursors. Multilineage dysplasia is common and often fulfills diagnostic criteria for MDS, which is closely associated with both anemia and thrombocytopenia (6, 38). Moreover, the chronic systemic inflammation sustained by aberrant myeloid clones can further suppress erythropoiesis and megakaryopoiesis through inflammatory cytokine-mediated mechanisms, aggravating cytopenias (36, 38).

Figure 1 provides a graphical representation of the predicted probability of VEXAS syndrome among patients diagnosed with Schnitzler's syndrome, according to the presence of thrombocytopenia, lymphadenopathy, or both variables, in relation to increasing age. This figure is also intended to serve a practical purpose, providing clinicians with a visual tool to estimate the probability that a patient presenting with features consistent with Schnitzler's syndrome may instead be affected by VEXAS syndrome.

The main limitation of this study lies in the non-exhaustiveness of the variables included in the statistical analysis. This is since the AIDA Network registries for Schnitzler's syndrome and VEXAS syndrome were designed differently based on the unique characteristics of each pathology (17, 18, 21, 22). Specifically, the increase in the mean corpuscular value (MCV) in the complete blood count is a rather common finding in VEXAS syndrome but not in Schnitzler's syndrome, and as such, the AIDA Network registry for Schnitzler's syndrome does not include this information. Similarly, certain clinical features are included in the VEXAS registry but not in the Schnitzler registry, such as the presence of chondritis, which is relatively frequent among patients with VEXAS syndrome (1, 6). It is precisely the presence of chondritis and MCV elevation that should be considered in the identification of VEXAS patients among those with suspected Schnitzler's syndrome, in addition to the factors identified in this study. Another important consideration is the response to anti-IL-1 and anti-IL-6 biotechnological treatments, which is likely to be more frequently complete among patients with Schnitzler's syndrome than among those with VEXAS syndrome (14–20). However, in this regard, an inferential statistical analysis was not feasible due to the very limited number of patients treated with these biotechnological agents. Of note, the study was restricted to male subjects to ensure a homogeneous and representative cohort. However, the differential diagnostic challenge addressed in this study should also be extended to female patients as soon as an adequate sample size becomes available. Finally, the search for potential MYD88 mutations was not performed in the patients with Schnitzler's syndrome included in this study; such an analysis could have provided additional insights to further refine the differential diagnosis

In conclusion, this study provides a comparative framework that highlights specific clinical, and laboratory features distinguishing VEXAS syndrome from Schnitzler's syndrome in male patients with cutaneous involvement. Specifically, patients with lymphadenopathy, thrombocytopenia, anemia, particularly in older age, and in the absence of leucocytosis, are more likely to be affected by VEXAS syndrome. These distinctions are crucial for clinicians when evaluating patients with autoinflammatory symptoms and may support the consideration of genetic testing for *UBA1* mutations in appropriate clinical contexts.

## Data Availability

The raw data supporting the conclusions of this article will be made available by the authors, without undue reservation. Requests to access these datasets should be directed to the corresponding author: Luca Cantarini, MD, PhD, Research Center of Systemic Autoinflammatory Diseases and Behçet's Disease Clinics, Department of Medical Sciences, Surgery and Neurosciences, University of Siena, cantariniluca@hotmail.com.
